# Supplementary material for: Exploring Spatial Heterogeneity of Immune Cells in Nasopharyngeal Cancer
Source: Cancers (Basel). 2023 Apr 5;15(7):2165. doi: 10.3390/cancers15072165 (PMC10093565; doi:10.3390/cancers15072165)
Supplement: Supplementary file 1 [file cancers-15-02165-s001.zip › cancers-2284050-supplementary.pdf]

# Supplementary Figures and Tables

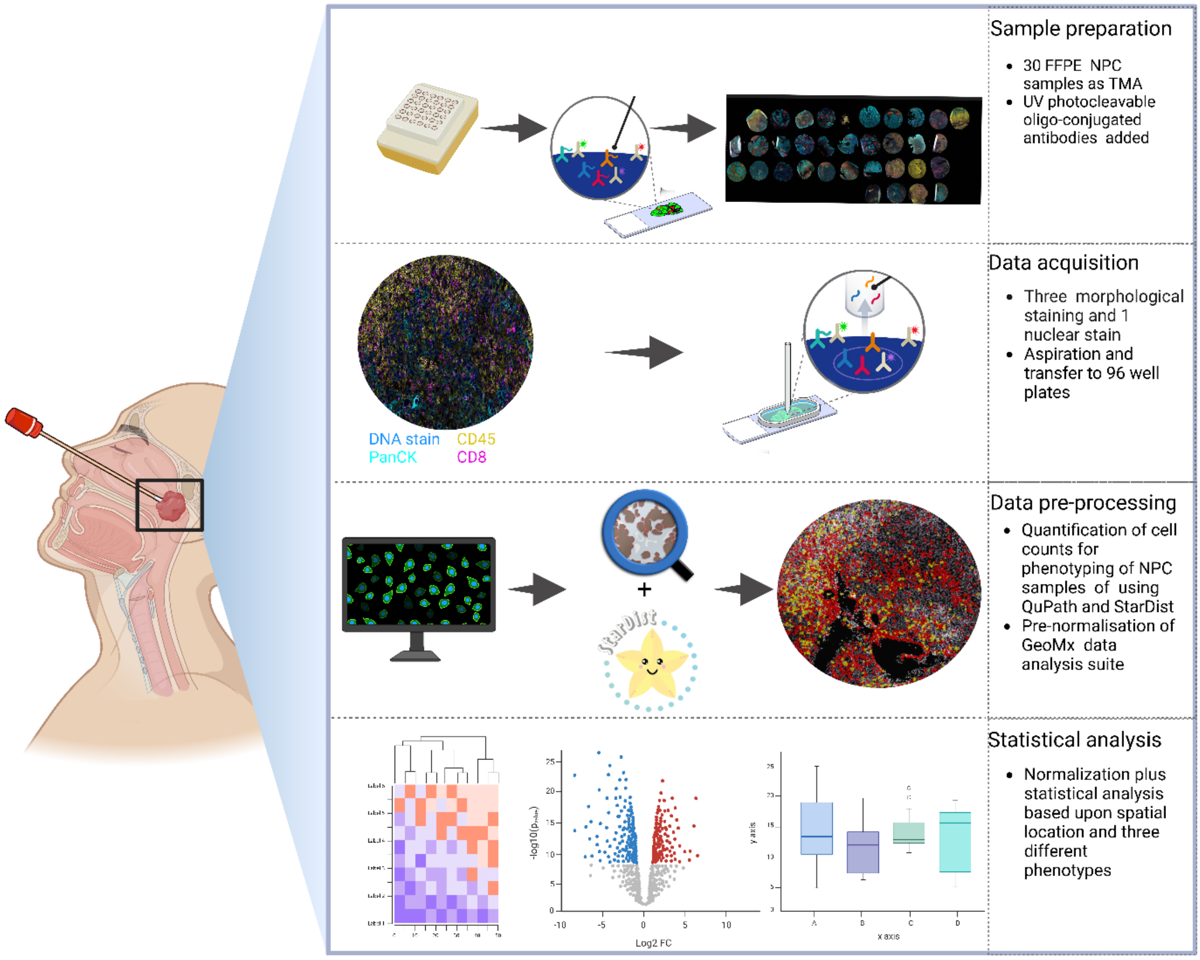


**Figure S1. Experimental setup**. Tissue morphology for the 30 FFPE NPC samples was investigated using PanCK, CD45, CD8, and nuclear staining. ROIs were selected and further profiled with a 43-plex oligo-conjugated antibody cocktail using the GeoMx® DSP system. Protein expression profiles were further analysed based on their spatial distribution in the three tumor immune phenotypes ('inflamed', 'immune-excluded', and 'desert').

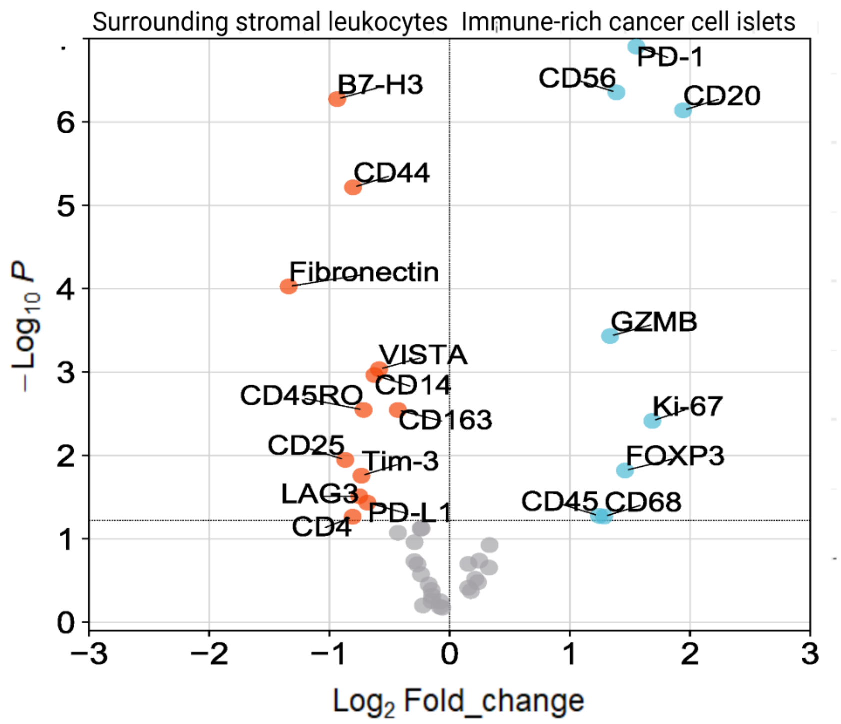


**Figure S2. Two-group comparison in areas of interest**. Volcano plot showing the differentially expressed proteins between the regions 'immune-rich cancer cell islets' and 'surrounding stromal leukocytes'. The values are based upon adjusted p-values, (Mann-Whitney test, Benjamini, Krieger, and Yekutieli correction, adjusted p-value<0.05).

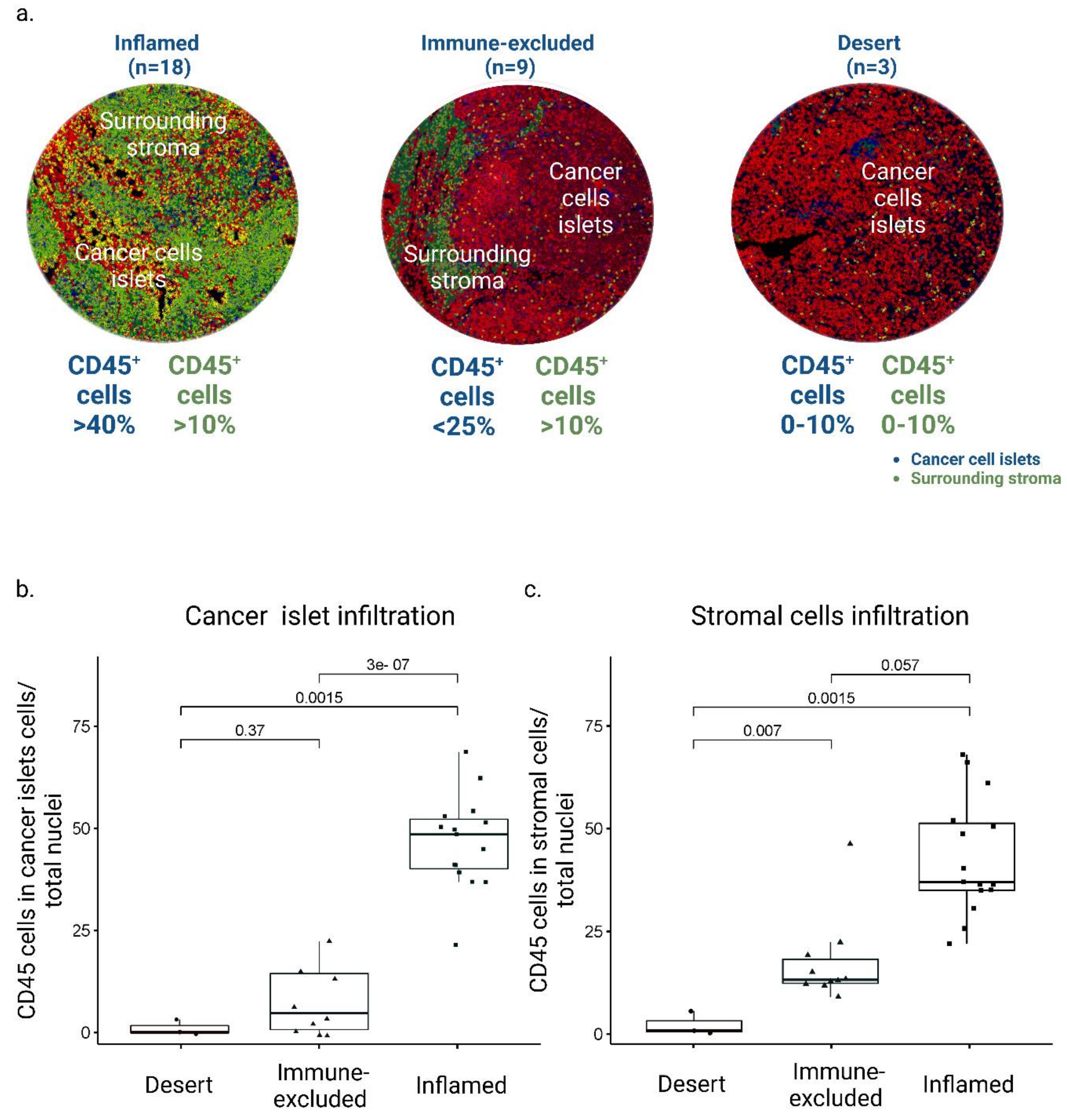


**Figure S3. Quantification of CD45$^{+}$ cells in NPC biopsies**. (**a**). Quantification of the CD45$^{+}$ and CD8$^{+}$ cells correlating with the phenotypes. The text below the figures depicts the percentages on which the classification was based. Box plots showing the percentage of CD45$^{+}$ cells in (**b**) the 'immune-rich cancer cell islets' and (**c**) 'surrounding stromal leukocytes' regions, respectively, within the three phenotypes ('inflamed', 'immune-excluded', and 'desert') (Kruskal-Wallis test, p-value<0.05).

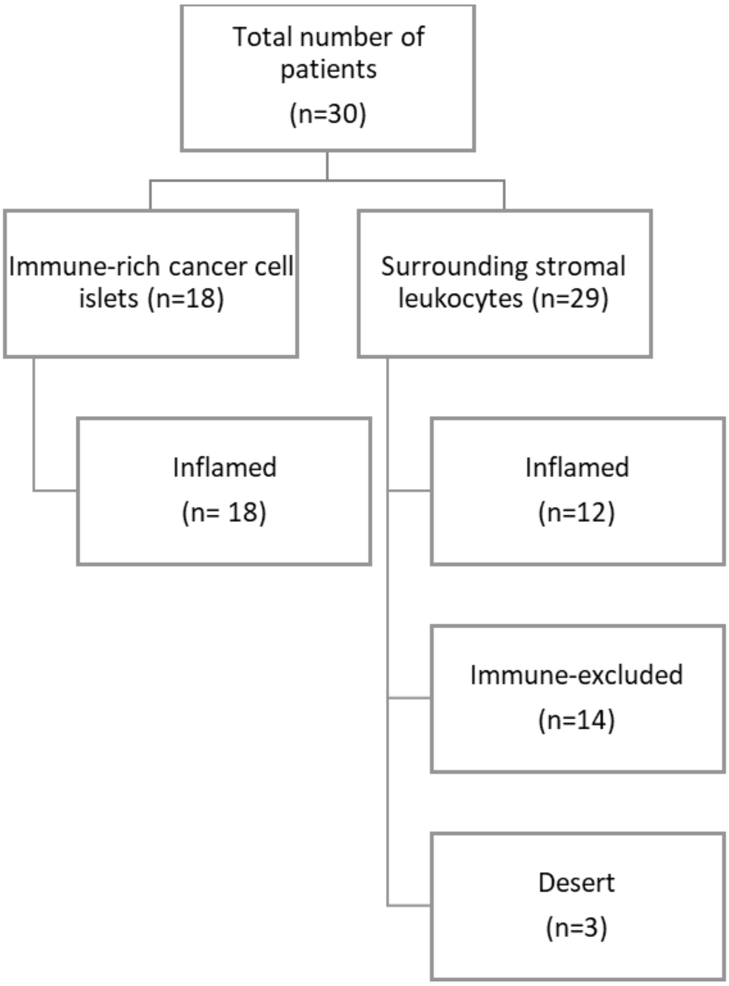


**Figure S4**. **Distribution of obtained areas of interest**. Different regions ('immune-rich cancer cell islets' and surrounding stromal segments') and their further division as seen in the identified phenotypes ('inflamed', 'immune-excluded' and 'desert').

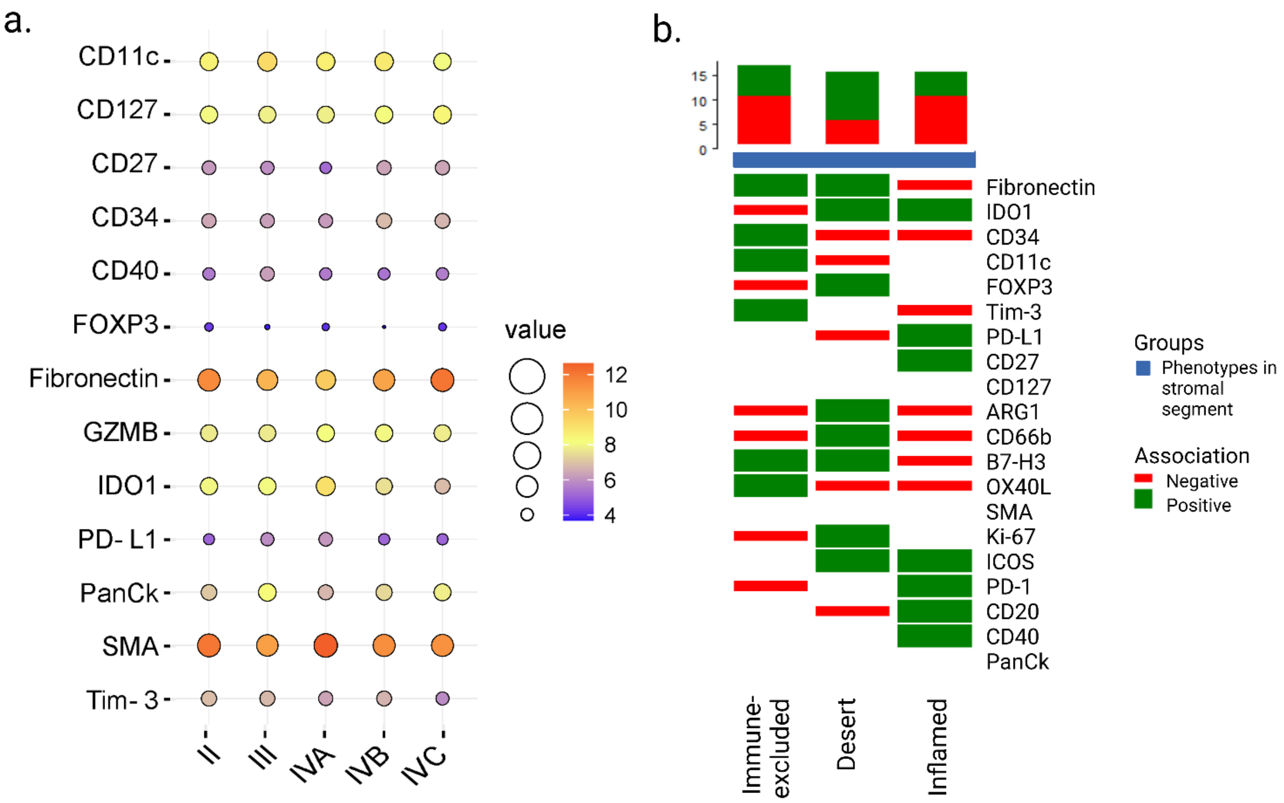


**Figure S5. Differential expression in TNM stages**. (**a**). Balloon plots showing average expression of significant protein markers in different TNM stages. The balloon's size and color correlate with each protein's expression within different clinical stages. (**b**). On an Oncoprint map, the most significant stromal regions' proteins associated with TNM staging among the three phenotypes: 'inflamed,' 'immune-excluded,' and 'desert', are represented.

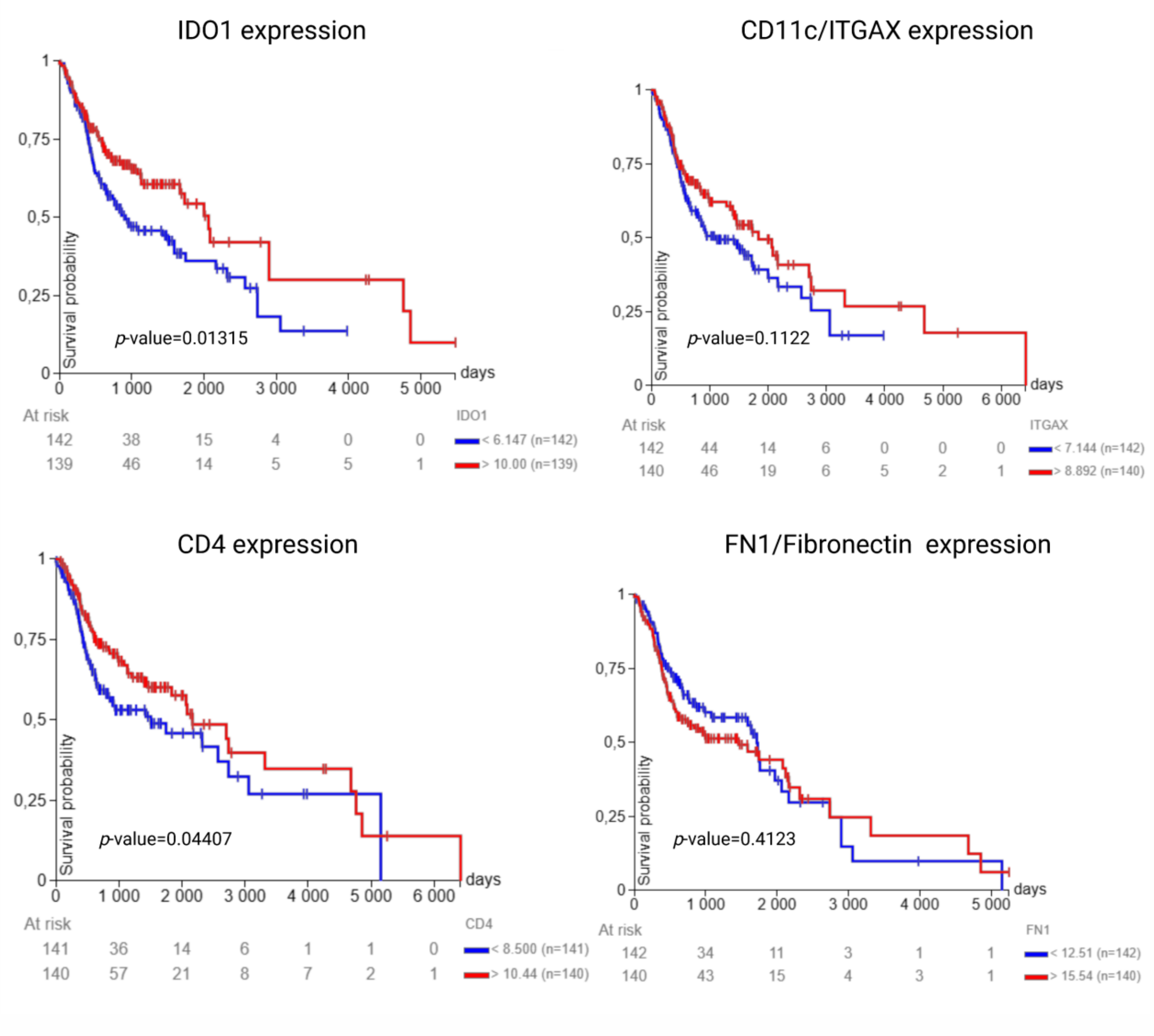


**Figure S6**. **Survival analysis using the TCGA HNSC dataset**. Kaplan Meier plots demonstrating the survival analysis based on gene expression of IDO1, CD11c/ITGAX, CD4, and Fibronectin/FN1 from the TCGA HNSC cohort. The groups were divided based on quartile differences, where the group in red represents the 4th quartile expression and the group in blue represents the 1st quartile.

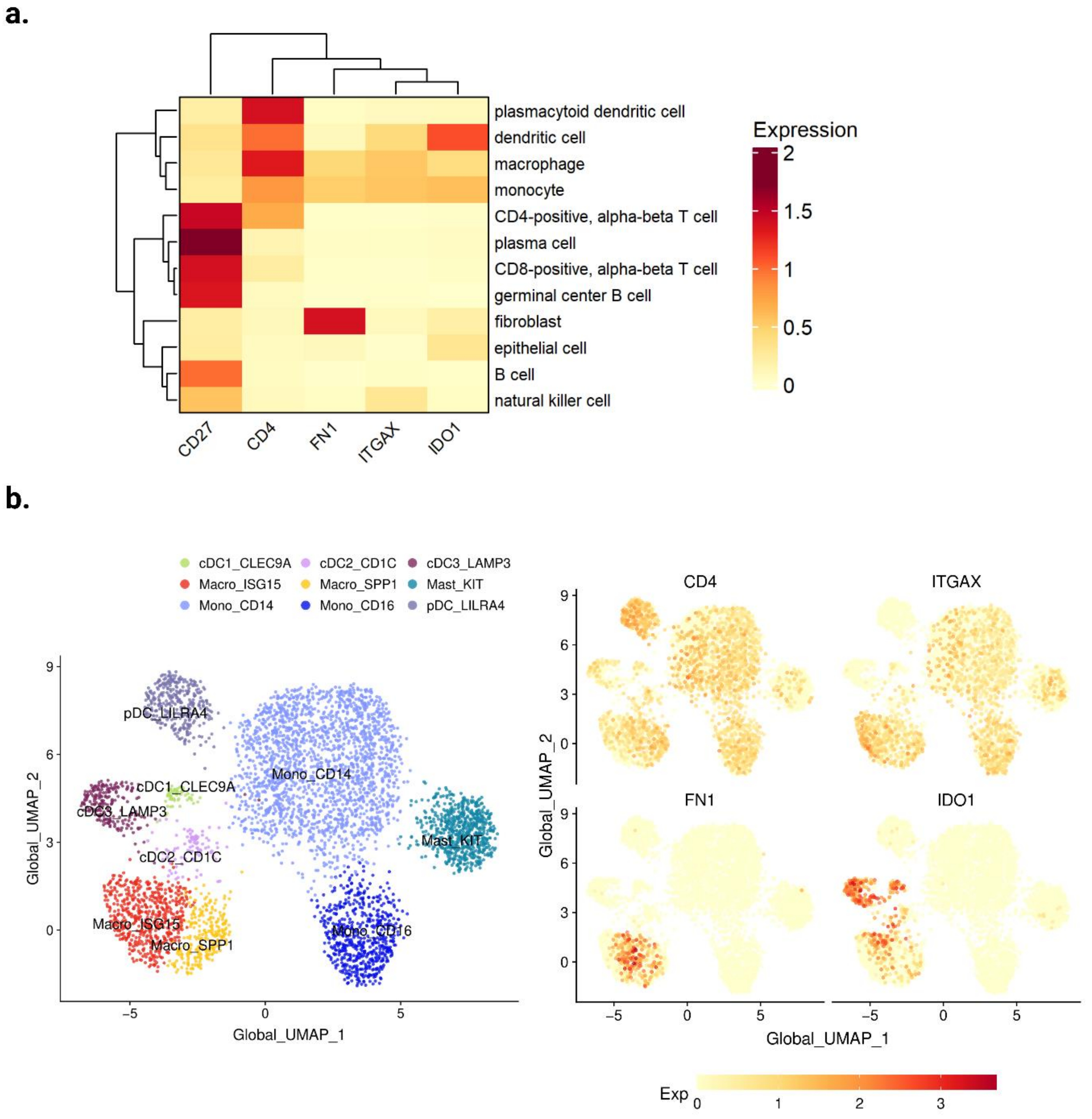


**Figure S7. Differential expression in TNM stages**. (**a**). Balloon plots showing average expression of significant protein markers in different TNM stages. The balloon's size and color correlate with each protein's expression within different clinical stages. (**b**). On an Oncoprint map, the most significant stromal regions' proteins associated with TNM staging among the three phenotypes: 'inflamed,' 'immune-excluded,' and 'desert', are represented.

**Table S1:** Clinicopathological characteristics of the cohort used for DSP analysis.

| | **Patient characteristics** | **No. of patients (n=30)** |
|---|---|---|
| Sex | Female/Male | 10/20 |
| Age Median | 57 years | |
| EBV status | Negative/Positive | 4/26 |
| Tumor stage (TNM-7) | I/II/III/IVA/IVB/IVC | 2/3/5/7/7/5 |
| Disease-specific mortality | Alive/Deceased | 26/4 |

**Table S2:** Protein profiling panels included in the DSP analysis.

| **Panels** | **Proteins** |
|---|---|
| Immune cell profiling core | PD-1, CD68, HLA-DR, Ki-67, Beta-2-microglobulin, CD11c, CD20, CD3, CD4, CD45, CD56, CD8, CTLA4, GZMB, PD-L1, PanCK, SMA, Fibronectin |
| Immune-oncology drug target module | 4-1BB, LAG3, OX40L, Tim-3, VISTA, ARG1, B7-H3, IDO1, STING, GITR |
| Immune activation status module | CD127, CD25, CD80, ICOS, PD-L2, CD40, CD44, CD27 |
| Immune cell typing module | CD45RO, FOXP3, CD34, CD66b, FAP-alpha, CD14, CD163 |

**Table S3**: Average expression values of DE markers by TNM stages associated with Figure S4a.

| Protein markers | Stage II | Stage III | Stage IVA | Stage IVB | Stage IVC |
|---|---|---|---|---|---|
| CD11c | 8.567133 | 9.2302 | 8.64545714 | 8.77455714 | 8.06038 |
| CD127 | 8.042133 | 7.8629 | 7.85697143 | 8.10445714 | 8.4376 |
| CD27 | 6.1626 | 5.9374 | 5.25081429 | 6.36157143 | 6.40662 |
| CD34 | 6.427667 | 6.276 | 6.18667143 | 6.83295714 | 6.69566 |
| CD40 | 5.543833 | 6.27012 | 5.54591429 | 5.38925714 | 5.59814 |
| Fibronectin | 11.562 | 10.35106 | 9.67524286 | 10.8241 | 12.1148 |
| FOXP3 | 4.3069 | 3.78368 | 4.07895714 | 3.68227143 | 4.18768 |
| GZMB | 7.7489 | 7.73156 | 8.11188571 | 8.01465714 | 7.78162 |
| IDO1 | 7.996933 | 8.06104 | 9.08818571 | 7.56065714 | 6.84828 |
| PanCK | 7.082233 | 8.27256 | 6.74314286 | 7.39724286 | 7.82662 |
| PD-L1 | 5.078367 | 5.87858 | 6.10664286 | 5.149 | 5.12592 |
| SMA | 12.06367 | 10.955 | 12.6378571 | 11.417 | 11.3492 |
| Tim-3 | 6.890967 | 6.788 | 6.3753 | 6.68394286 | 5.8624 |

TNM staging according to UICC's TNM classification system, 7th version

**Table S4**: Adjusted p-values of significant proteins obtained after cox regression analysis

| | Overall-survival | | Overall-survival (7-year) | | Disease-free survival | | Disease-free survival (7-year) | |
|---|---|---|---|---|---|---|---|---|
| Protein | Univariate analysis | Multivariate analysis | Univariate analysis | Multivariate analysis | Univariate analysis | Multivariate analysis | Univariate analysis | Multivariate analysis |
| CD11c | 0.014 | 0.002 | 0.029 | 0.028 | 0.0365 | 0.04 | 0.026 | 0.026 |
| Fibronectin | 0.029 | 0.03 | 0.003 | 0.003 | 0.0001 | 0.002 | 0.0021 | 0.002 |
| IDO1 | 0.003 | 0.023 | 0.0042 | 0.0039 | 0.0035 | 0.04 | 0.004 | 0.0039 |
| CD4 | 0.022 | 0.001 | 0.014 | 0.0138 | 0.023 | 0.03 | 0.011 | 0.01 |
| CD27 | 0.004 | 0.03 | 0.022 | 0.0218 | 0.038 | 0.04 | 0.023 | 0.022 |

The values represent p-values associated with hazard ratio of significant proteins for each survival parameter.

**Table S5**: Kaplan-Meier analysis of the significant proteins obtained via cox regression analysis by survival parameters.

| Protein | Overall-survival | Overall-survival (7-year) | Disease-free survival | Disease-free survival (7-year) |
|---|---|---|---|---|
| CD11c | 0.028 | 0.0024 | 0.0001 | 0.0044 |
| Fibronectin | 0.035 | 0.0026 | 0.0023 | 0.007 |
| IDO1 | 0.045 | 0.0079 | 0.0051 | 0.019 |
| CD4 | 0.037 | 0.043 | 0.036 | 0.03 |
| CD27 | 0.34 | 0.38 | 0.3 | 0.38 |

The values represent p-values associated with each survival parameter.
